# SEX AND ETHNICITY-RELATED DISPARITIES IN ALZHEIMER’S DISEASE AMONG RESIDENTS OF SKILLED NURSING FACILITIES

**DOI:** 10.1093/geroni/igad104.0572

**Published:** 2023-12-21

**Authors:** Arseniy Yashkin, Igor Akushevich

**Affiliations:** Duke University, Durham, North Carolina, United States; Duke University, Durham, North Carolina, United States

## Abstract

Residents of nursing homes represent a special subset of the population characterized by increased vulnerability and higher rates of Alzheimer’s Disease (AD). Nursing home ethnic composition does not reflect that of the general population with minorities more likely to be concentrated in facilities with serious deficiencies, and to have lower health status at time of admission. Significant ethnic disparities in quality-of-care aspects such as hospitalizations, preventive care, and the management of chronic conditions exist and these patterns extend to sex and ethnicity-specific subgroups of individuals with AD. However, there is a paucity of knowledge on the actual size of sex and ethnicity-related disparities in AD within this high-risk population. In this study we used 100% of all nursing home resident evaluations (N=23,080,518; Male:40.66%; Female:59.34%; White: 77.29%; Black:11.62%; Other:3.80%; Hispanic:4.97%; Asian:1.81%; Pacific Islander:0.15%; Native American Native:0.39%), 1999-2012, from the Minimum Dataset to assess sex and ethnicity-specific differences in age-specific prevalence of AD. Total AD prevalence increases sharply (numbers are prevalence proportions with 95% confidence intervals in parentheses) from 1.79 (1.75-1.84) at age 60 to 13.45 (13.39-13.51) at 85. Notable statistically significant sex and ethnicity-related disparities were present at all ages. For example, at age 85 the prevalence of AD was for: i)Males: 11.34 (11.24-11.44); ii)Females: 14.53 (14.45-14.62); iii)Whites: 13.21 (13.14-13.28); iv)Blacks: 15.32 (15.09-15.55); v)Other/Unknown: 9.75 (9.42-10.07); vi)Hispanics: 18.17 (17.82-18.52); vii)Asians: 11.30 (10.87-11.72); viii)Pacific Islanders: 10.33 (8.65-12.01); ix)North American Natives: 11.78 (10.62-12.94). In depth analysis of the causes, time-trends, and geographic distribution of these disparities is needed.

